# Observation of deuteron and antideuteron formation from resonance-decay nucleons

**DOI:** 10.1038/s41586-025-09775-5

**Published:** 2025-12-10

**Authors:** The ALICE Collaboration, S. Acharya, A. Agarwal, G. Aglieri Rinella, L. Aglietta, M. Agnello, N. Agrawal, Z. Ahammed, S. Ahmad, S. U. Ahn, I. Ahuja, A. Akindinov, V. Akishina, M. Al-Turany, D. Aleksandrov, B. Alessandro, H. M. Alfanda, R. Alfaro Molina, B. Ali, A. Alici, N. Alizadehvandchali, A. Alkin, J. Alme, G. Alocco, T. Alt, A. R. Altamura, I. Altsybeev, J. R. Alvarado, M. N. Anaam, C. Andrei, N. Andreou, A. Andronic, E. Andronov, V. Anguelov, F. Antinori, P. Antonioli, N. Apadula, H. Appelshäuser, C. Arata, S. Arcelli, R. Arnaldi, J. G. M. C. A. Arneiro, I. C. Arsene, M. Arslandok, A. Augustinus, R. Averbeck, D. Averyanov, M. D. Azmi, H. Baba, A. Badalà, J. Bae, Y. Bae, Y. W. Baek, X. Bai, R. Bailhache, Y. Bailung, R. Bala, A. Baldisseri, B. Balis, S. Bangalia, Z. Banoo, V. Barbasova, F. Barile, L. Barioglio, M. Barlou, B. Barman, G. G. Barnaföldi, L. S. Barnby, E. Barreau, V. Barret, L. Barreto, K. Barth, E. Bartsch, N. Bastid, S. Basu, G. Batigne, D. Battistini, B. Batyunya, D. Bauri, J. L. Bazo Alba, I. G. Bearden, P. Becht, D. Behera, I. Belikov, A. D. C. Bell Hechavarria, F. Bellini, R. Bellwied, S. Belokurova, L. G. E. Beltran, Y. A. V. Beltran, G. Bencedi, A. Bensaoula, S. Beole, Y. Berdnikov, A. Berdnikova, L. Bergmann, L. Bernardinis, L. Betev, P. P. Bhaduri, T. Bhalla, A. Bhasin, B. Bhattacharjee, S. Bhattarai, L. Bianchi, J. Bielčk, J. Bielčková, A. P. Bigot, A. Bilandzic, A. Binoy, G. Biro, S. Biswas, N. Bize, D. Blau, M. B. Blidaru, N. Bluhme, C. Blume, F. Bock, T. Bodova, J. Bok, L. Boldizsár, M. Bombara, P. M. Bond, G. Bonomi, H. Borel, A. Borissov, A. G. Borquez Carcamo, E. Botta, Y. E. M. Bouziani, D. C. Brandibur, L. Bratrud, P. Braun-Munzinger, M. Bregant, M. Broz, G. E. Bruno, V. D. Buchakchiev, M. D. Buckland, D. Budnikov, H. Buesching, S. Bufalino, P. Buhler, N. Burmasov, Z. Buthelezi, A. Bylinkin, S. A. Bysiak, J. C. Cabanillas Noris, M. F. T. Cabrera, H. Caines, A. Caliva, E. Calvo Villar, J. M. M. Camacho, P. Camerini, M. T. Camerlingo, F. D. M. Canedo, S. Cannito, S. L. Cantway, M. Carabas, F. Carnesecchi, L. A. D. Carvalho, J. Castillo Castellanos, M. Castoldi, F. Catalano, S. Cattaruzzi, R. Cerri, I. Chakaberia, P. Chakraborty, S. Chandra, S. Chapeland, M. Chartier, S. Chattopadhay, M. Chen, T. Cheng, C. Cheshkov, D. Chiappara, V. Chibante Barroso, D. D. Chinellato, F. Chinu, E. S. Chizzali, J. Cho, S. Cho, P. Chochula, Z. A. Chochulska, D. Choudhury, S. Choudhury, P. Christakoglou, C. H. Christensen, P. Christiansen, T. Chujo, M. Ciacco, C. Cicalo, G. Cimador, F. Cindolo, M. R. Ciupek, G. Clai, F. Colamaria, J. S. Colburn, D. Colella, A. Colelli, M. Colocci, M. Concas, G. Conesa Balbastre, Z. Conesa del Valle, G. Contin, J. G. Contreras, M. L. Coquet, P. Cortese, M. R. Cosentino, F. Costa, S. Costanza, P. Crochet, M. M. Czarnynoga, A. Dainese, G. Dange, M. C. Danisch, A. Danu, P. Das, S. Das, A. R. Dash, S. Dash, A. De Caro, G. de Cataldo, J. de Cuveland, A. De Falco, D. De Gruttola, N. DeMarco, C. De Martin, S. DePasquale, R. Deb, R. DelGrande, L. Dello Stritto, G. G. A. de Souza, P. Dhankher, D. DiBari, M. Di Costanzo, A. Di Mauro, B. Di Ruzza, B. Diab, R. A. Diaz, Y. Ding, J. Ditzel, R. Divià, Ø. Djuvsland, U. Dmitrieva, A. Dobrin, B. Dönigus, J. M. Dubinski, A. Dubla, P. Dupieux, N. Dzalaiova, T. M. Eder, R. J. Ehlers, F. Eisenhut, R. Ejima, D. Elia, B. Erazmus, F. Ercolessi, B. Espagnon, G. Eulisse, D. Evans, S. Evdokimov, L. Fabbietti, M. Faggin, J. Faivre, F. Fan, W. Fan, T. Fang, A. Fantoni, M. Fasel, G. Feofilov, A. Fernández Téllez, L. Ferrandi, M. B. Ferrer, A. Ferrero, C. Ferrero, A. Ferretti, V. J. G. Feuillard, V. Filova, D. Finogeev, F. M. Fionda, F. Flor, A. N. Flores, S. Foertsch, I. Fokin, S. Fokin, U. Follo, E. Fragiacomo, E. Frajna, H. Fribert, U. Fuchs, N. Funicello, C. Furget, A. Furs, T. Fusayasu, J. J. Gaardhøje, M. Gagliardi, A. M. Gago, T. Gahlaut, C. D. Galvan, S. Gami, D. R. Gangadharan, P. Ganoti, C. Garabatos, J. M. Garcia, T. García Chávez, E. Garcia-Solis, S. Garetti, C. Gargiulo, P. Gasik, H. M. Gaur, A. Gautam, M. B. Gay Ducati, M. Germain, R. A. Gernhaeuser, C. Ghosh, M. Giacalone, G. Gioachin, S. K. Giri, P. Giubellino, P. Giubilato, A. M. C. Glaenzer, P. Glässel, E. Glimos, V. Gonzalez, P. Gordeev, M. Gorgon, K. Goswami, S. Gotovac, V. Grabski, L. K. Graczykowski, E. Grecka, A. Grelli, C. Grigoras, V. Grigoriev, S. Grigoryan, O. S. Groettvik, F. Grosa, J. F. Grosse-Oetringhaus, R. Grosso, D. Grund, N. A. Grunwald, R. Guernane, M. Guilbaud, K. Gulbrandsen, J. K. Gumprecht, T. Gündem, T. Gunji, J. Guo, W. Guo, A. Gupta, R. Gupta, R. Gupta, K. Gwizdziel, L. Gyulai, C. Hadjidakis, F. U. Haider, S. Haidlova, M. Haldar, H. Hamagaki, Y. Han, B. G. Hanley, R. Hannigan, J. Hansen, J. W. Harris, A. Harton, M. V. Hartung, H. Hassan, D. Hatzifotiadou, P. Hauer, L. B. Havener, E. Hellbär, H. Helstrup, M. Hemmer, T. Herman, S. G. Hernandez, G. Herrera Corral, S. Herrmann, K. F. Hetland, B. Heybeck, H. Hillemanns, B. Hippolyte, I. P. M. Hobus, F. W. Hoffmann, B. Hofman, A. Horzyk, Y. Hou, P. Hristov, P. Huhn, L. M. Huhta, T. J. Humanic, A. Hutson, D. Hutter, M. C. Hwang, R. Ilkaev, M. Inaba, M. Ippolitov, A. Isakov, T. Isidori, M. S. Islam, S. Iurchenko, M. Ivanov, M. Ivanov, V. Ivanov, K. E. Iversen, M. Jablonski, B. Jacak, N. Jacazio, P. M. Jacobs, S. Jadlovska, J. Jadlovsky, S. Jaelani, C. Jahnke, M. J. Jakubowska, M. A. Janik, S. Ji, S. Jia, T. Jiang, A. A. P. Jimenez, S. Jin, F. Jonas, D. M. Jones, J. M. Jowett, J. Jung, M. Jung, A. Junique, A. Jusko, J. Kaewjai, P. Kalinak, A. Kalweit, A. Karasu Uysal, N. Karatzenis, O. Karavichev, T. Karavicheva, E. Karpechev, M. J. Karwowska, U. Kebschull, M. Keil, B. Ketzer, J. Keul, S. S. Khade, A. M. Khan, S. Khan, A. Khanzadeev, Y. Kharlov, A. Khatun, A. Khuntia, Z. Khuranova, B. Kileng, B. Kim, C. Kim, D. J. Kim, D. Kim, E. J. Kim, G. Kim, H. Kim, J. Kim, J. Kim, J. Kim, M. Kim, S. Kim, T. Kim, K. Kimura, S. Kirsch, I. Kisel, S. Kiselev, A. Kisiel, J. L. Klay, J. Klein, S. Klein, C. Klein-Bösing, M. Kleiner, T. Klemenz, A. Kluge, C. Kobdaj, R. Kohara, T. Kollegger, A. Kondratyev, N. Kondratyeva, J. Konig, S. A. Konigstorfer, P. J. Konopka, G. Kornakov, M. Korwieser, S. D. Koryciak, C. Koster, A. Kotliarov, N. Kovacic, V. Kovalenko, M. Kowalski, V. Kozhuharov, G. Kozlov, I. Králik, A. Kravčáková, L. Krcal, M. Krivda, F. Krizek, K. Krizkova Gajdosova, C. Krug, M. Krüger, D. M. Krupova, E. Kryshen, V. Kučera, C. Kuhn, P. G. Kuijer, T. Kumaoka, D. Kumar, L. Kumar, N. Kumar, S. Kumar, S. Kundu, M. Kuo, P. Kurashvili, A. B. Kurepin, A. Kuryakin, S. Kushpil, V. Kuskov, M. Kutyla, A. Kuznetsov, M. J. Kweon, Y. Kwon, S. L. LaPointe, P. LaRocca, A. Lakrathok, M. Lamanna, S. Lambert, A. R. Landou, R. Langoy, P. Larionov, E. Laudi, L. Lautner, R. A. N. Laveaga, R. Lavicka, R. Lea, H. Lee, I. Legrand, G. Legras, A. M. Lejeune, T. M. Lelek, R. C. Lemmon, I. León Monzón, M. M. Lesch, P. Lévai, M. Li, P. Li, X. Li, B. E. Liang-Gilman, J. Lien, R. Lietava, I. Likmeta, B. Lim, H. Lim, S. H. Lim, S. Lin, V. Lindenstruth, C. Lippmann, D. Liskova, D. H. Liu, J. Liu, G. S. S. Liveraro, I. M. Lofnes, C. Loizides, S. Lokos, J. Lömker, X. Lopez, E. López Torres, C. Lotteau, P. Lu, W. Lu, Z. Lu, F. V. Lugo, J. Luo, G. Luparello, M. A. T. Johnson, Y. G. Ma, M. Mager, M. Mahlein, A. Maire, E. M. Majerz, M. V. Makariev, M. Malaev, G. Malfattore, N. M. Malik, N. Malik, S. K. Malik, D. Mallick, N. Mallick, G. Mandaglio, S. K. Mandal, A. Manea, V. Manko, A. K. Manna, F. Manso, G. Mantzaridis, V. Manzari, Y. Mao, R. W. Marcjan, G. V. Margagliotti, A. Margotti, A. Marn, C. Markert, P. Martinengo, M. I. Martínez, G. Martínez García, M. P. P. Martins, S. Masciocchi, M. Masera, A. Masoni, L. Massacrier, O. Massen, A. Mastroserio, L. Mattei, S. Mattiazzo, A. Matyja, F. Mazzaschi, M. Mazzilli, Y. Melikyan, M. Melo, A. Menchaca-Rocha, J. E. M. Mendez, E. Meninno, A. S. Menon, M. W. Menzel, M. Meres, L. Micheletti, D. Mihai, D. L. Mihaylov, A. U. Mikalsen, K. Mikhaylov, N. Minafra, D. Miśkowiec, A. Modak, B. Mohanty, M. Mohisin Khan, M. A. Molander, M. M. Mondal, S. Monira, C. Mordasini, D. A. Moreira DeGodoy, I. Morozov, A. Morsch, T. Mrnjavac, V. Muccifora, S. Muhuri, A. Mulliri, M. G. Munhoz, R. H. Munzer, H. Murakami, L. Musa, J. Musinsky, J. W. Myrcha, N. B. Sundstrom, B. Naik, A. I. Nambrath, B. K. Nandi, R. Nania, E. Nappi, A. F. Nassirpour, V. Nastase, A. Nath, N. F. Nathanson, C. Nattrass, K. Naumov, M. N. Naydenov, A. Neagu, L. Nellen, R. Nepeivoda, S. Nese, N. Nicassio, B. S. Nielsen, E. G. Nielsen, S. Nikolaev, V. Nikulin, F. Noferini, S. Noh, P. Nomokonov, J. Norman, N. Novitzky, J. Nystrand, M. R. Ockleton, M. Ogino, S. Oh, A. Ohlson, V. A. Okorokov, J. Oleniacz, C. Oppedisano, A. Ortiz Velasquez, J. Otwinowski, M. Oya, K. Oyama, S. Padhan, D. Pagano, G. Paić, S. Paisano-Guzmán, A. Palasciano, I. Panasenko, S. Panebianco, P. Panigrahi, C. Pantouvakis, H. Park, J. Park, S. Park, J. E. Parkkila, Y. Patley, R. N. Patra, P. Paudel, B. Paul, H. Pei, T. Peitzmann, X. Peng, M. Pennisi, S. Perciballi, D. Peresunko, G. M. Perez, Y. Pestov, V. Petrov, M. Petrovici, S. Piano, M. Pikna, P. Pillot, O. Pinazza, L. Pinsky, C. Pinto, S. Pisano, M. Płoskoń, M. Planinic, D. K. Plociennik, M. G. Poghosyan, B. Polichtchouk, S. Politano, N. Poljak, A. Pop, S. Porteboeuf-Houssais, I. Y. Pozos, K. K. Pradhan, S. K. Prasad, S. Prasad, R. Preghenella, F. Prino, C. A. Pruneau, I. Pshenichnov, M. Puccio, S. Pucillo, L. Quaglia, A. M. K. Radhakrishnan, S. Ragoni, A. Rai, A. Rakotozafindrabe, N. Ramasubramanian, L. Ramello, C. O. Ramírez-Álvarez, M. Rasa, S. S. Räsänen, R. Rath, M. P. Rauch, I. Ravasenga, K. F. Read, C. Reckziegel, A. R. Redelbach, K. Redlich, C. A. Reetz, H. D. Regules-Medel, A. Rehman, F. Reidt, H. A. Reme-Ness, K. Reygers, A. Riabov, V. Riabov, R. Ricci, M. Richter, A. A. Riedel, W. Riegler, A. G. Riffero, M. Rignanese, C. Ripoli, C. Ristea, M. V. Rodriguez, M. Rodríguez Cahuantzi, K. Røed, R. Rogalev, E. Rogochaya, D. Rohr, D. Röhrich, S. Rojas Torres, P. S. Rokita, G. Romanenko, F. Ronchetti, D. Rosales Herrera, E. D. Rosas, K. Roslon, A. Rossi, A. Roy, S. Roy, N. Rubini, J. A. Rudolph, D. Ruggiano, R. Rui, P. G. Russek, R. Russo, A. Rustamov, E. Ryabinkin, Y. Ryabov, A. Rybicki, L. C. V. Ryder, J. Ryu, W. Rzesa, B. Sabiu, S. Sadhu, S. Sadovsky, J. Saetre, S. Saha, B. Sahoo, R. Sahoo, D. Sahu, P. K. Sahu, J. Saini, K. Sajdakova, S. Sakai, S. Sambyal, D. Samitz, I. Sanna, T. B. Saramela, D. Sarkar, P. Sarma, V. Sarritzu, V. M. Sarti, M. H. P. Sas, S. Sawan, E. Scapparone, J. Schambach, H. S. Scheid, C. Schiaua, R. Schicker, F. Schlepper, A. Schmah, C. Schmidt, M. O. Schmidt, M. Schmidt, N. V. Schmidt, A. R. Schmier, J. Schoengarth, R. Schotter, A. Schröter, J. Schukraft, K. Schweda, G. Scioli, E. Scomparin, J. E. Seger, Y. Sekiguchi, D. Sekihata, M. Selina, I. Selyuzhenkov, S. Senyukov, J. J. Seo, D. Serebryakov, L. Serkin, L. Šerkšnytė, A. Sevcenco, T. J. Shaba, A. Shabetai, R. Shahoyan, A. Shangaraev, B. Sharma, D. Sharma, H. Sharma, M. Sharma, S. Sharma, T. Sharma, U. Sharma, A. Shatat, O. Sheibani, K. Shigaki, M. Shimomura, S. Shirinkin, Q. Shou, Y. Sibiriak, S. Siddhanta, T. Siemiarczuk, T. F. Silva, D. Silvermyr, T. Simantathammakul, R. Simeonov, B. Singh, B. Singh, K. Singh, R. Singh, R. Singh, S. Singh, V. K. Singh, V. Singhal, T. Sinha, B. Sitar, M. Sitta, T. B. Skaali, G. Skorodumovs, N. Smirnov, R. J. M. Snellings, E. H. Solheim, C. Sonnabend, J. M. Sonneveld, F. Soramel, A. B. Soto-Hernandez, R. Spijkers, I. Sputowska, J. Staa, J. Stachel, I. Stan, T. Stellhorn, S. F. Stiefelmaier, D. Stocco, I. Storehaug, N. J. Strangmann, P. Stratmann, S. Strazzi, A. Sturniolo, C. P. Stylianidis, A. A. P. Suaide, C. Suire, A. Suiu, M. Sukhanov, M. Suljic, R. Sultanov, V. Sumberia, S. Sumowidagdo, L. H. Tabares, S. F. Taghavi, J. Takahashi, G. J. Tambave, Z. Tang, J. Tanwar, J. D. Tapia Takaki, N. Tapus, L. A. Tarasovicova, M. G. Tarzila, A. Tauro, A. Tavira García, G. Tejeda Muñoz, L. Terlizzi, C. Terrevoli, D. Thakur, S. Thakur, M. Thogersen, D. Thomas, A. Tikhonov, N. Tiltmann, A. R. Timmins, M. Tkacik, A. Toia, R. Tokumoto, S. Tomassini, K. Tomohiro, N. Topilskaya, M. Toppi, V. V. Torres, A. Trifiró, T. Triloki, A. S. Triolo, S. Tripathy, T. Tripathy, S. Trogolo, V. Trubnikov, W. H. Trzaska, T. P. Trzcinski, C. Tsolanta, R. Tu, A. Tumkin, R. Turrisi, T. S. Tveter, K. Ullaland, B. Ulukutlu, S. Upadhyaya, A. Uras, M. Urioni, G. L. Usai, M. Vaid, M. Vala, N. Valle, L. V. R. van Doremalen, M. van Leeuwen, C. A. vanVeen, R. J. G. van Weelden, D. Varga, Z. Varga, P. Vargas Torres, M. Vasileiou, A. Vasiliev, O. Vázquez Doce, O. Vazquez Rueda, V. Vechernin, P. Veen, E. Vercellin, R. Verma, R. Vértesi, M. Verweij, L. Vickovic, Z. Vilakazi, O. Villalobos Baillie, A. Villani, A. Vinogradov, T. Virgili, M. M. O. Virta, A. Vodopyanov, B. Volkel, M. A. Völkl, S. A. Voloshin, G. Volpe, B. vonHaller, I. Vorobyev, N. Vozniuk, J. Vrláková, J. Wan, C. Wang, D. Wang, Y. Wang, Y. Wang, Z. Wang, A. Wegrzynek, F. Weiglhofer, S. C. Wenzel, J. P. Wessels, P. K. Wiacek, J. Wiechula, J. Wikne, G. Wilk, J. Wilkinson, G. A. Willems, B. Windelband, M. Winn, J. R. Wright, W. Wu, Y. Wu, K. Xiong, Z. Xiong, R. Xu, A. Yadav, A. K. Yadav, Y. Yamaguchi, S. Yang, S. Yang, S. Yano, E. R. Yeats, J. Yi, Z. Yin, I.-K. Yoo, J. H. Yoon, H. Yu, S. Yuan, A. Yuncu, V. Zaccolo, C. Zampolli, F. Zanone, N. Zardoshti, P. Závada, M. Zhalov, B. Zhang, C. Zhang, L. Zhang, M. Zhang, M. Zhang, S. Zhang, X. Zhang, Y. Zhang, Z. Zhang, M. Zhao, V. Zherebchevskii, Y. Zhi, D. Zhou, Y. Zhou, J. Zhu, S. Zhu, Y. Zhu, S. C. Zugravel, N. Zurlo

**Affiliations:** 1 INFN Sezione di Bari, Bari, Italy; 2 Variable Energy Cyclotron Centre, Homi Bhabha National Institute, Kolkata, India; 3 European Organization for Nuclear Research (CERN), Geneva, Switzerland; 4 Dipartimento di Fisica dell’Università and Sezione, INFN, Turin, Italy; 5 Dipartimento DISAT del Politecnico and Sezione, INFN, Turin, Italy; 6 Dipartimento di Fisica e Astronomia dell’Università and Sezione, INFN, Bologna, Italy; 7 Department of Physics, Aligarh Muslim University, Aligarh, India; 8 Korea Institute of Science and Technology Information, Daejeon, Republic of Korea; 9 Faculty of Science, P.J. Šafárik University, Košice, Slovak Republic; 10 Affiliated with an institute formerly covered by a cooperation agreement with CERN, Geneva, Switzerland; 11 Frankfurt Institute for Advanced Studies, Johann Wolfgang Goethe-Universität Frankfurt, Frankfurt, Germany; 12 Research Division and ExtreMe Matter Institute EMMI, GSI Helmholtzzentrum für Schwerionenforschung, Darmstadt, Germany; 13 INFN Sezione di Torino, Turin, Italy; 14 Central China Normal University, Wuhan, China; 15 Instituto de Fsica, Universidad Nacional Autónoma de México, Mexico City, Mexico; 16 University of Houston, Houston, TX USA; 17 Sungkyunkwan University, Suwon City, Republic of Korea; 18 Department of Physics and Technology, University of Bergen, Bergen, Norway; 19 Institut für Kernphysik, Johann Wolfgang Goethe-Universität Frankfurt, Frankfurt, Germany; 20 Physik Department, Technische Universität München, Munich, Germany; 21 High Energy Physics Group, Universidad Autónoma de Puebla, Puebla, Mexico; 22 Horia Hulubei National Institute of Physics and Nuclear Engineering, Bucharest, Romania; 23 University of Derby, Derby, UK; 24 Institut für Kernphysik, Universität Münster, Münster, Germany; 25 Physikalisches Institut, Ruprecht-Karls-Universität Heidelberg, Heidelberg, Germany; 26 INFN Sezione di Padova, Padova, Italy; 27 INFN Sezione di Bologna, Bologna, Italy; 28 Lawrence Berkeley National Laboratory, Berkeley, CA USA; 29 Laboratoire de Physique Subatomique et de Cosmologie, Université Grenoble-Alpes, CNRS-IN2P3, Grenoble, France; 30 Universidade de São Paulo (USP), São Paulo, Brazil; 31 Department of Physics, University of Oslo, Oslo, Norway; 32 Yale University, New Haven, CT USA; 33 University of Tokyo, Tokyo, Japan; 34 INFN Sezione di Catania, Catania, Italy; 35 Gangneung-Wonju National University, Gangneung, Republic of Korea; 36 University of Science and Technology of China, Hefei, China; 37 Indian Institute of Technology Indore, Indore, India; 38 Physics Department, University of Jammu, Jammu, India; 39 IRFU Départment de Physique Nucléaire (DPhN), Centre d’Etudes de Saclay (CEA), Université Paris-Saclay, Saclay, France; 40 AGH University of Krakow, Cracow, Poland; 41 University of Kansas, Lawrence, KS USA; 42 Dipartimento Interateneo di Fisica, ‘M. Merlin’ and Sezione INFN, Bari, Italy; 43 National and Kapodistrian University of Athens, School of Science, Department of Physics, National and Kapodistrian University of Athens, Athens, Greece; 44 Department of Physics, Gauhati University, Guwahati, India; 45 HUN-REN Wigner Research Centre for Physics, Budapest, Hungary; 46 SUBATECH, IMT Atlantique, Nantes Université, CNRS-IN2P3, Nantes, France; 47 Université Clermont Auvergne, CNRS/IN2P3 LPC, Clermont-Ferrand, France; 48 Department of Physics, Lund University, Lund, Sweden; 49 Affiliated with an international laboratory covered by a cooperation agreement with CERN, Geneva, Switzerland; 50 Indian Institute of Technology Bombay (IIT), Mumbai, India; 51 Sección Fsica, Departamento de Ciencias, Pontificia Universidad Católica del Perú, Lima, Peru; 52 Niels Bohr Institute, University of Copenhagen, Copenhagen, Denmark; 53 Université de Strasbourg, CNRS, IPHC UMR 7178, Strasbourg, France; 54 Universidad Autónoma de Sinaloa, Culiacán, Mexico; 55 Dipartimento di Fisica dell’Università and Sezione, INFN, Trieste, Italy; 56 Physics Department, Panjab University, Chandigarh, India; 57 Faculty of Nuclear Sciences and Physical Engineering, Czech Technical University in Prague, Prague, Czech Republic; 58 Nuclear Physics Institute of the Czech Academy of Sciences, Husinec-Řež, Czech Republic; 59 Department of Physics and Centre for Astroparticle Physics and Space Science (CAPSS), Bose Institute, Kolkata, India; 60 Oak Ridge National Laboratory, Oak Ridge, TN USA; 61 Department of Physics, Pusan National University, Pusan, Republic of Korea; 62 Università di Brescia, Brescia, Italy; 63 INFN Sezione di Pavia, Pavia, Italy; 64 Institute of Space Science (ISS), Bucharest, Romania; 65 Politecnico di Bari and Sezione INFN, Bari, Italy; 66 Faculty of Physics, Sofia University, Sofia, Bulgaria; 67 Nuclear Physics Group, STFC Daresbury Laboratory, Daresbury, UK; 68 Stefan Meyer Institut für Subatomare Physik (SMI), Vienna, Austria; 69 iThemba LABS, National Research Foundation, Somerset West, South Africa; 70 University of the Witwatersrand, Johannesburg, South Africa; 71 The Henryk Niewodniczanski Institute of Nuclear Physics, Polish Academy of Sciences, Cracow, Poland; 72 Dipartimento di Fisica ‘E.R. Caianiello’, Università degli Studi di Salerno and Gruppo Collegato INFN, Salerno, Italy; 73 Universitatea Nationala de Stiinta si Tehnologie Politehnica Bucuresti, Bucharest, Romania; 74 Warsaw University of Technology, Warsaw, Poland; 75 University of Liverpool, Liverpool, UK; 76 Fudan University, Shanghai, China; 77 Institut de Physique des 2 Infinis de Lyon, Université de Lyon, CNRS/IN2P3, Lyon, France; 78 Dipartimento di Fisica e Astronomia dell’Università and Sezione INFN, Padova, Italy; 79 Inha University, Incheon, Republic of Korea; 80 Saha Institute of Nuclear Physics, Homi Bhabha National Institute, Kolkata, India; 81 Nikhef National institute for Subatomic Physics, Amsterdam, The Netherlands; 82 University of Tsukuba, Tsukuba, Japan; 83 INFN Sezione di Cagliari, Cagliari, Italy; 84 School of Physics and Astronomy, University of Birmingham, Birmingham, UK; 85 IJCLab, Université Paris-Saclay, CNRS/IN2P3, Orsay, France; 86 Università del Piemonte Orientale, Vercelli, Italy; 87 Universidade Federal do ABC, Santo Andre, Brazil; 88 Dipartimento di Fisica, Università di Pavia, Pavia, Italy; 89 Dipartimento di Fisica dell’Università and Sezione, INFN, Cagliari, Italy; 90 Department of Physics, University of California Berkeley, Berkeley CA, USA; 91 Università degli Studi di Foggia, Foggia, Italy; 92 Faculty of Mathematics, Physics and Informatics, Comenius University Bratislava, Bratislava, Slovak Republic; 93 Physics Program and International Institute for Sustainability with Knotted Chiral Meta Matter (WPI-SKCM2), Hiroshima University, Hiroshima, Japan; 94 Laboratori Nazionali di Frascati, INFN, Frascati, Italy; 95 The University of Texas at Austin, Austin, TX USA; 96 Sezione di Trieste, INFN, Trieste, Italy; 97 Saga University, Saga, Japan; 98 National Institute of Science Education and Research, Homi Bhabha National Institute, Jatni, India; 99 Chicago State University, Chicago, IL USA; 100 Instituto de Física, Universidade Federal do Rio Grande do Sul (UFRGS), Porto Alegre, Brazil; 101 University of Tennessee, Knoxville, TN USA; 102 Wayne State University, Detroit, MI USA; 103 Faculty of Electrical Engineering, Mechanical Engineering and Naval Architecture, University of Split, Split, Croatia; 104 Institute for Gravitational and Subatomic Physics (GRASP), Utrecht University/Nikhef, Utrecht, Netherlands; 105 A.I. Alikhanyan National Science Laboratory (Yerevan Physics Institute) Foundation, Yerevan, Armenia; 106 China Nuclear Data Center, China Institute of Atomic Energy, Beijing, China; 107 Nagasaki Institute of Applied Science, Nagasaki, Japan; 108 Yonsei University, Seoul, Republic of Korea; 109 University of Jyväskylä, Jyväskylä, Finland; 110 Helmholtz-Institut für Strahlen- und Kernphysik, Rheinische Friedrich-Wilhelms-Universität Bonn, Bonn, Germany; 111 Faculty of Technology, Environmental and Social Sciences, Bergen, Norway; 112 Centro de Investigación y de Estudios Avanzados (CINVESTAV), Mexico City and Mérida, Mexico; 113 Fachbereich Informatik und Mathematik, Johann-Wolfgang-Goethe Universität Frankfurt Institut für Informatik, Frankfurt, Germany; 114 Ohio State University, Columbus OH, USA; 115 Technical University of Košice, Košice, Slovak Republic; 116 National Research and Innovation Agency - BRIN, Jakarta, Indonesia; 117 Universidade Estadual de Campinas (UNICAMP), Campinas, Brazil; 118 Instituto de Ciencias Nucleares, Universidad Nacional Autónoma de México, Mexico City, Mexico; 119 Suranaree University of Technology, Nakhon Ratchasima, Thailand; 120 Institute of Experimental Physics, Slovak Academy of Sciences, Košice, Slovak Republic; 121 Yildiz Technical University, Istanbul, Turkey; 122 Jeonbuk National University, Jeonju, Republic of Korea; 123 Department of Physics, Sejong University, Seoul, Republic of Korea; 124 California Polytechnic State University, San Luis Obispo CA, USA; 125 Physics Department, Faculty of Science, University of Zagreb, Zagreb, Croatia; 126 National Centre for Nuclear Research, Warsaw, Poland; 127 Dipartimento di Fisica e Astronomia dell’Università and Sezione INFN, Catania, Italy; 128 University of South-Eastern Norway, Kongsberg, Norway; 129 Centro de Aplicaciones Tecnológicas y Desarrollo Nuclear (CEADEN), Havana, Cuba; 130 Dipartimento di Scienze MIFT, Università di Messina, Messina, Italy; 131 Helsinki Institute of Physics (HIP), Helsinki, Finland; 132 Chungbuk National University, Cheongju, Republic of Korea; 133 China University of Geosciences, Wuhan, China; 134 Creighton University, Omaha, NE USA; 135 National Nuclear Research Center, Baku, Azerbaijan; 136 Institute of Physics, Homi Bhabha National Institute, Bhubaneswar, India; 137 Physikalisches Institut, Eberhard-Karls-Universität Tübingen, Tübingen, Germany; 138 Nara Women’s University (NWU), Nara, Japan; 139 Bogolyubov Institute for Theoretical Physics, National Academy of Sciences of Ukraine, Kiev, Ukraine; 140 Institute of Physics of the Czech Academy of Sciences, Prague, Czech Republic; 141 Present Address: Max-Planck-Institut fur Physik, Munich, Germany; 142 Present Address: Italian National Agency for New Technologies, Energy and Sustainable Economic Development (ENEA), Bologna, Italy; 143 Present Address: Instituto de Fisica da Universidade de São Paulo, São Paulo, Brazil; 144 Present Address: Dipartimento DET del Politecnico di Torino, Turin, Italy; 145 Present Address: Department of Applied Physics, Aligarh Muslim University, Aligarh, India; 146 Present Address: Institute of Theoretical Physics, University of Wroclaw, Wrocław, Poland; 147 Present Address: Facultad de Ciencias, Universidad Nacional Autónoma de México, Mexico City, Mexico

**Keywords:** Experimental nuclear physics, Experimental particle physics

## Abstract

High-energy hadronic collisions generate environments characterized by temperatures above 100 MeV (refs. ^1,2^), about 100,000 times hotter than the centre of the Sun. At present, it is therefore unclear how light (anti)nuclei with mass number *A* of a few units, such as the deuteron, ^3^He or ^4^He, each bound by only a few MeV, can emerge from these collisions^3,4^. Here, the ALICE Collaboration reports that deuteron–pion momentum correlations in proton–proton (pp) collisions provide model-independent evidence that about 90% of the observed (anti)deuterons are produced in nuclear reactions^5^ following the decay of short-lived resonances, such as the Δ(1232). These findings, obtained by the ALICE Collaboration at the Large Hadron Collider, resolve a gap in our understanding of nucleosynthesis in ultrarelativistic hadronic collisions. Apart from offering insights on how (anti)nuclei are formed in hadronic collisions, the results can be used in the modelling of the production of light and heavy nuclei in cosmic rays^6^ and dark-matter decays^7,8^.

## Abstract (web summary)

The ALICE Collaboration provides details on the microscopic mechanism that ends up creating antideuteron in high-energy proton–proton collisions at the Large Hadron Collider.

## Main

Nuclear physicists have long been intrigued by the microscopic mechanism behind the formation of light nuclei and antinuclei in hadron–hadron collisions^3,4^. In ultrarelativistic heavy-ion collisions with energies per nucleon up to a few TeV (10^12^ eV), the study of particle production is directly connected to the confinement of colour charge in colour-neutral hadrons. These collisions produce a quark–gluon plasma, a state in which quarks and gluons are deconfined, and as the system evolves, they bind into hadrons and light (anti)nuclei^1,9^. As the binding energies of light (anti)nuclei are substantially lower (2.23 MeV for deuteron, 7.72 MeV for ^3^He; ref. ^10^) than the average kinetic energy of hadrons in these energetic collisions (of the order of 100 MeV; ref. ^1^), the question is about both the formation of loosely bound nuclei and their survival through the hadronic phase that follows the hadronization of the quark–gluon plasma. This issue is also relevant in ultrarelativistic proton–proton (pp) and proton–nucleus (p–A) collisions, in which the formation of a quark–gluon plasma remains under experimental and theoretical scrutiny and average kinetic energies above 100 MeV can still be achieved^2^.

The study of (anti)nucleus formation in hadronic collisions is of importance in astrophysics. On one front, the precise composition of ultrahigh-energy (>PeV) cosmic rays, particularly their heavier elements (*A* > 50) component, remains an open question^6^. A microscopic modelling of nucleus formation in ultrarelativistic hadron collisions is an essential ingredient for understanding the composition of these cosmic rays and for uncovering the origin of particle acceleration mechanisms in the Universe^11^. On another front, antinuclei formation—whether from cosmic-ray interactions with the interstellar medium or as potential products of dark-matter decay—plays a pivotal part in indirect searches for dark matter^7,8,12^. Experimental investigations into the microscopic processes underlying light nucleus and antinucleus formation thus offer a dual benefit: they advance the knowledge of the strong interaction in the non-perturbative regime and provide the quantitative framework needed to decode the spectra of cosmic rays and their origins.

The yields of nuclei such as deuterons (p–n bound system), ^3^H (p–n–n), ^3^He (p–p–n), ^4^He (p–p–n–n), $\genfrac{}{}{0ex}{}{3}{\Lambda }{\rm{H}}$ (Λ–p–n) and their corresponding antinuclei have been precisely measured at the Relativistic Heavy Ion Collider in Au–Au collisions at centre-of-mass energies per nucleon pair (√*s*_NN_) across an energy range of 7.7–200 GeV (refs. ^13–16^) and at the Large Hadron Collider (LHC) for pp collisions at √*s* ranging from 0.9 TeV to 13 TeV, as well as for p–Pb and Pb–Pb collisions at √*s*_NN_ = 2.76–8.16 TeV (refs. ^17–20^). Current understanding suggests that nuclei can be produced either through direct emission as multi-quark states following a collision, similar to other hadrons such as protons or pions, or through a secondary binding mechanism of nucleons.

Two types of models have been used to study these mechanisms. Statistical hadronization models describe the direct production and assume that hadrons and nuclei are directly emitted from a source in thermal and chemical equilibrium, with abundances determined by the particle mass, the system temperature, volume and quantum number conservation^9,21,22^. This work uses the canonical statistical model (CSM)^23^, which is better suited for pp collisions. Although CSMs predict yields effectively, they do not provide insights into the microscopic mechanisms driving (anti)nucleus formation. By contrast, coalescence models^12,24–27^ emulate binding mechanisms and they assume that (anti)nucleons form independently before binding to create (anti)nuclei. This approach incorporates microscopic parameters, such as the spatial proximity of nucleons alongside their strong interactions, allowing for a satisfactory description of yields and momentum distributions^25,28^.

Microscopic calculations implemented in event generators for heavy-ion collisions^5,29^ include pion-catalysed reactions—both formation and disintegration (for example, π + p + n ⇔ π + d)—and successfully describe measured nuclear yields. The important aspect of these models is that a third body, such as a meson, aids the binding process by carrying away the excess energy.

Overall a direct experimental evidence for the microscopic mechanisms of (anti)nucleus formation remains unknown. Femtoscopy provides a complementary approach by examining pion–(anti)deuteron (π–d) momentum correlations and offers direct insights into the microscopic processes underlying (anti)deuteron formation. This technique has been effectively used by the ALICE Collaboration to study various hadron pairs produced in pp and p–Pb collisions at the LHC, see for example, ref. ^30^ and the references therein, shedding light on their residual strong interactions.

Using π–d femtoscopy correlations, the study presented in this paper shows, in a model-independent manner, that (anti)deuterons are formed following the decay of strong resonances, such as the Δ(1232) (hereafter Δ). Considering the possible contribution to all produced resonances, we estimate that 88.9 ± 6.3% of the observed (anti)deuterons are generated through binding processes. These findings resolve a longstanding puzzle regarding the formation of light (anti)nuclei in collider experiments and provide a robust foundation for further modelling of nucleosynthesis from hadronic collisions, both in accelerators and in the Universe.

## Resonances and correlation function

As a bound state of a proton and a neutron, the deuteron may inherit correlations developed by its constituent nucleons during the evolution of the collision. The study of π–d correlations offers a sensitive probe of this process. If deuterons are produced thermally alongside pions, any correlation signal between them would arise solely from final-state interactions. By contrast, if deuterons form through the coalescence of nucleons originating from the decay of an intermediate resonance, the resulting π–d correlation function could retain the signatures of that resonance.

The correlation function *C*(k*) is the key experimental observable, and k* is the single-particle momentum in the pair rest frame (PRF). Experimentally, $C({k}^{* })={\mathcal{N}}\,[{N}_{{\rm{same}}}({k}^{* })/{N}_{{\rm{mixed}}}({k}^{* })]$, where *N*_same_(k*) (same-event sample) is the distribution of relative momenta between the π–d pair measured for pions and deuterons stemming from the same collision^31^. Equivalently, *N*_mixed_(k*) (mixed-event sample) is an uncorrelated reference obtained by building the distribution through the combination of pions and deuterons originating from different collisions. Finally, ${\mathcal{N}}$ is a normalization factor ensuring the proper convergence of *C*(k*) to unity at large k*. In the case of non-interacting particles, the correlation function is equal to unity for all k* as the relative momentum distribution is purely governed by the underlying single-particle phase space, which is the same for *N*_same_(k*) and *N*_mixed_(k*) distributions. An attractive interaction enhances the correlation function above unity at low k* ≲ 200 MeV *c*^−1^, whereas a repulsive interaction leads to a depletion below unity. A resonance that decays into the hadron pair of interest would produce a peak in the k* spectrum.

**Fig. 1:**
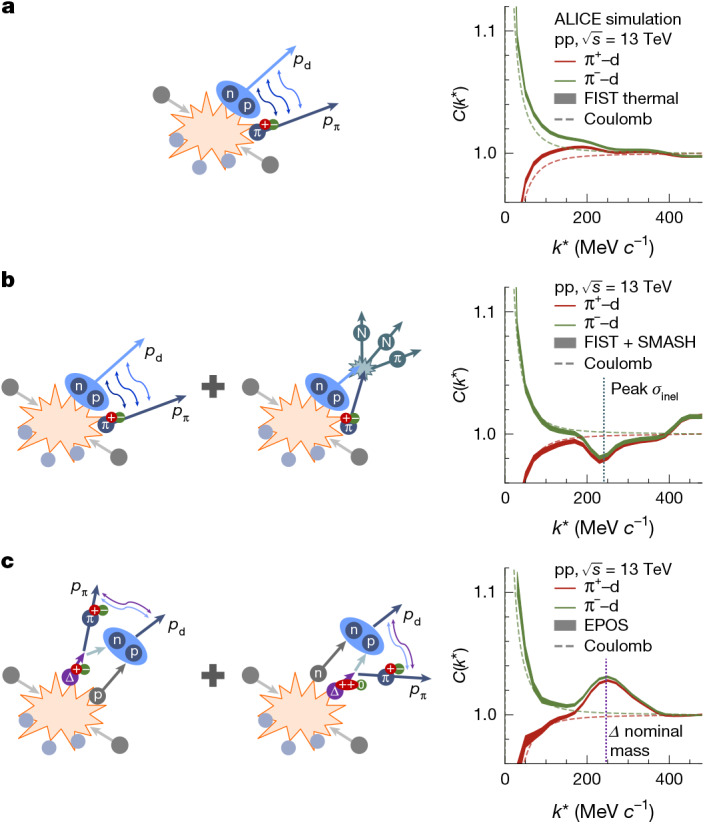
(Anti)deuteron production scenarios. Illustration of three scenarios for deuteron production and interaction with pions (left) and the resulting π^±^–d correlation functions (right). All scenarios include Coulomb attraction between π^−^–d (green curves) and Coulomb repulsion between the π^+^–d (red curves). The dashed lines always show the correlation function using Coulomb interaction. **a**,**b**, Thermally produced deuterons with only Coulomb (**a**) and Coulomb + elastic + inelastic (**b**) interactions, respectively. **c**, Deuteron formation by nuclear binding following Δ-resonance decays. All the simulations include the charge conjugates (${{\rm{\pi }}}^{+}-{\rm{d}}\equiv {{\rm{\pi }}}^{+}-{\rm{d}}\oplus {{\rm{\pi }}}^{-}-\overline{{\rm{d}}}$ and ${{\rm{\pi }}}^{-}-{\rm{d}}\equiv {{\rm{\pi }}}^{-}-{\rm{d}}\oplus {{\rm{\pi }}}^{+}-\overline{{\rm{d}}}$). The bandwidths corresponds to the statistical uncertainties of the models.

Figure 1 shows three scenarios of (anti)deuteron production mechanisms and interactions and the resulting π^±^–d correlations. Simulations were performed to quantify the effects of the different production scenarios, and details are provided in the Methods. All scenarios include repulsive (red curves) and attractive (green curves) Coulomb interactions for the π^+^–d and π^−^–d systems, respectively. The strong interaction contribution is minimal because of the small scattering parameters of the π^±^−d system and is hence neglected^32,33^. In the first two scenarios (Fig. 1a,b), directly produced deuterons and pions are considered. The simulation results shown on the right were obtained assuming that pions and (anti)deuterons are produced following a canonical statistical hadronization scheme, ThermalFIST^34^. The obtained correlation functions are multiplied by the Coulomb correlation function. The results indicate a depletion in the correlation function at low k* for π^+^–d and an enhancement for π^−^–d because of the Coulomb interaction.

In Fig. 1b, the elastic and inelastic scattering of pions and deuterons is considered. This is tested by using the hadronic transport model SMASH^35^ as an afterburner to ThermalFIST to simulate inelastic and elastic rescattering according to the experimental cross-sections. The elastic processes do not modify the shape of the correlation function, as both the incoming and outgoing π^±^–d pairs must conserve energy, ensuring that their relative momentum k* remains unchanged. The same holds for pseudo-elastic processes, in which an intermediate Δ resonance is formed, as in π + (pn) → pΔ → π + (pn).

By contrast, inelastic π^±^–d scattering leads to deuteron destruction, reducing the number of measurable pairs in the k* region in which the inelastic cross-section reaches its maximum. Both elastic and inelastic cross-sections peak at the nominal Δ mass (k* ≈ 240 MeV *c*^−1^), and the inelastic one is three times larger than the elastic contribution^36^. Figure 1b (right) shows the results of these simulations for the π^+^–d and π^−^–d cases. As expected, a depletion at the relative momentum k* ≈ 240 MeV *c*^−1^, corresponding to the peak of the inelastic cross-section, is observed.

In Fig. 1c, a deuteron forms when a primordial nucleon binds with one from a Δ decay. These resonances are very short-lived excited states of nucleons and decay after approximately 1.5 fm *c*^−1^ into π–nucleon pairs. Considering all charge states (Δ^++,+,0,−^), they are expected to contribute 43% of the nucleon yield in pp collisions at the LHC^34,37^. Measurements of the π^±^–p femtoscopy correlations by ALICE^38^ have already shown the presence of the Δ resonances, modified by rescattering and regeneration effects (Methods). For the formation of deuterons, the possible combinations include neutron–proton binding from Δ^++^ → π^+^–p or Δ^0^ → π^−^–p, proton–neutron binding from Δ^±^ → π^±^–n, and binding of two nucleons from separate Δ decays. This scenario was simulated by exploiting a state-of-the-art coalescence afterburner^25^ combined with the EPOS 3 event generator^39,40^. The latter accounts for resonance production and their decays, whereas the aforementioned rescattering and regeneration effects are not included in the simulations. The results are shown in the right panel, and a clear peak appears in correspondence with the Δ resonance nominal mass. The observed peak is due to the residual correlation between the pion and an (anti)nucleon from the Δ decay during the (anti)deuteron formation process.

The three patterns in the correlation function correspond to different physics scenarios and are easily distinguishable from one another. The observed patterns remain unchanged across different models: although model parametrization may shift or rescale the structures, their shapes are preserved. This constitutes a solid reference to proceed with the interpretation of the experimental data.

## Discussion

**Fig. 2:**
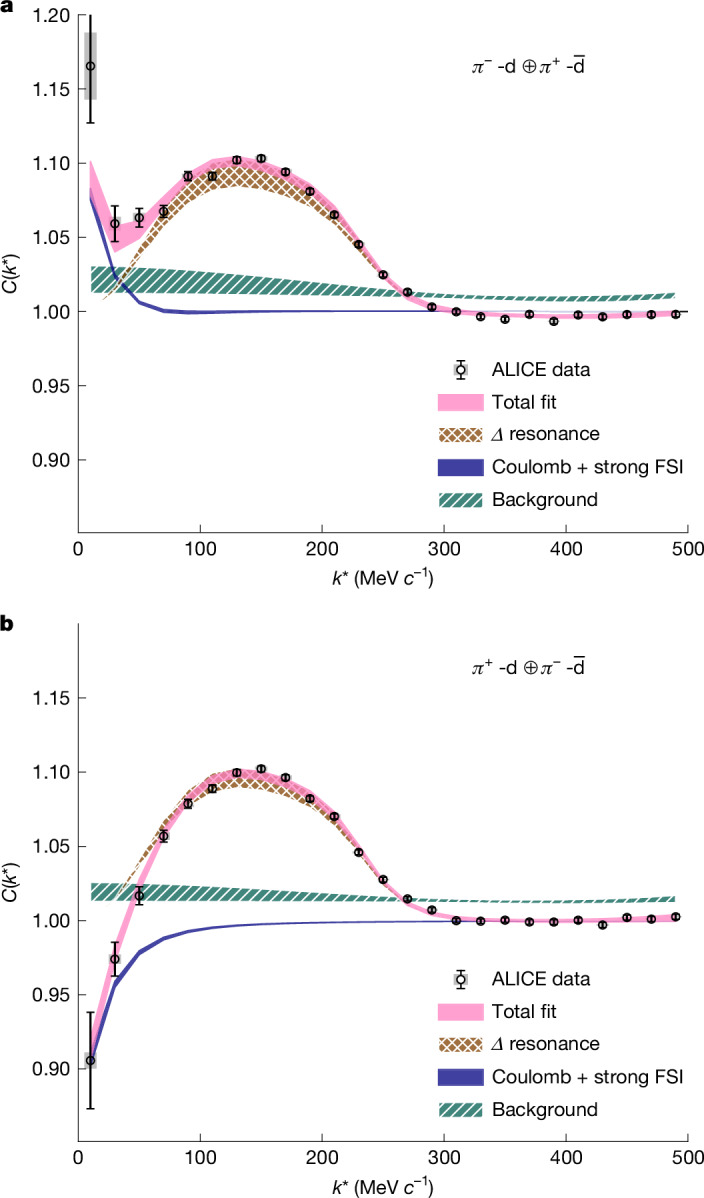
Experimental π^−^–d and π^+^–d correlation functions. The data are obtained from high-multiplicity pp collisions at √*s* = 13 TeV. **a**, The measured π^−^–d correlation function together with the corresponding fit function (magenta). The brown cross-hatched band represents contributions from the Δ resonance, the blue band denotes the Coulomb and strong FSI interactions, and the teal diagonally hatched band corresponds to the residual background. The widths of the bands indicate the fit uncertainty. **b**, In the same representation, the π^+^–d correlation function. However, the strong FSI interaction is neglected for this system. The *χ*^2^ per degree of freedom is 14/15 for both correlations.

The experimental π^+^–d and π^−^–d correlation functions have been measured in pp collisions at √*s* = 13 TeV. Charged pions (π^±^), deuteron (d) and antideuteron ($\overline{{\rm{d}}}$) tracks are reconstructed with the ALICE detector, and their momentum transverse to the beam direction (*p*_T_) is measured in the range *p*_T_ ∈ [0.14, 4.0] GeV *c*^−1^ for pions and *p*_T_ ∈ [0.8, 2.4]  GeV *c*^−1^ for deuterons. The excellent particle identification and tracking abilities of the ALICE detector provide samples of π^+^ (π^−^) and d ($\overline{{\rm{d}}}$) with a purity of 99% and 100%, respectively. Further details on the particle selection and evaluation of the systematic uncertainties are described in the Methods. After the selection of pions and (anti)deuterons, the correlation functions for pairs of particles (π^+^–d and π^−^–d) and their charge conjugates (${{\rm{\pi }}}^{+}-\overline{{\rm{d}}}$ and ${{\rm{\pi }}}^{-}-\overline{{\rm{d}}}$) are obtained. As the same interaction governs hadron–hadron and antihadron–antihadron pairs^31^, the sum of particles and antiparticles is considered (${{\rm{\pi }}}^{+}-{\rm{d}}\equiv {{\rm{\pi }}}^{+}-{\rm{d}}\oplus {{\rm{\pi }}}^{-}-\overline{{\rm{d}}}$ and ${{\rm{\pi }}}^{-}-{\rm{d}}\equiv {{\rm{\pi }}}^{-}-{\rm{d}}\oplus {{\rm{\pi }}}^{+}-\overline{{\rm{d}}}$) in the following. The resulting π^−^–d and π^+^–d correlation functions are shown by the open markers in Fig. 2a,b. The grey boxes around the markers represent the systematic uncertainties, and the vertical bars show the statistical uncertainties. The fit results for the π^−^–d and π^+^–d correlation functions are shown in Fig. 2a,b, respectively.

The measured π^±^–d correlation functions are modelled and fitted using a decomposition approach summarized by the relation ${C}_{{\rm{fit}}}({k}^{* })=\varepsilon ({k}^{* })\otimes B({k}^{* })[{\lambda }_{{\rm{gen}}}{C}_{{\rm{gen}}}({k}^{* })+(1-{\lambda }_{{\rm{gen}}})]$ (Methods). Here, *ε*(k*) represents a correction for momentum resolution effects, and *B*(k*) is a baseline accounting for residual background correlations. The latter arise mainly from non-primary pions produced in weak decays of long-lived resonances, as well as from secondary particles originating from interactions with the detector material. These contributions can mimic correlated pairs and must be accounted for in the modelling. The parameter *λ*_gen_ quantifies the fraction of genuine π^±^–d pairs, with the non-genuine component primarily arising from the feed-down of long-lived resonances into pions^41^ with a lifetime *τ* > 5 fm *c*^−1^. The term *C*_gen_(k*) denotes the corresponding genuine correlation function that contains Coulomb and strong interactions, alongside contributions from the Δ resonance. The interaction components are modelled using the CATS (Correlation Analysis Tool using the Schrödinger equation) framework^42^. Theoretically, $C({k}^{* })=\int {{\rm{d}}}^{3}{r}^{* }S({r}^{* })\times {| \psi ({{\bf{k}}}^{* },{{\bf{r}}}^{* })| }^{2}$, where r* is the relative distance (in the PRF) between the particles at the time of their effective emission, *ψ*(k*, r*) is the wavefunction of the pair relative motion, and *S*(r*) is the source function corresponding to the probability to emit the pair at a certain relative distance r* (ref. ^43^). Dedicated studies of the source function in pp collisions at √*s* = 13 TeV performed by the ALICE Collaboration showed a common emission source for all hadrons^38,41,44^. This source is typically modelled by a Gaussian function with a standard deviation (an effective size of the source) of *r*_eff_ ≈ 1.5 fm, obtained by accounting for the contribution of short-lived resonances (see Methods for details).

For the source, an effective Gaussian distribution with *r*_eff_ = 1.51 ± 0.12 fm was used (Methods). The real part of the π^−^–d potential is included in the fit. However, owing to the small scattering parameters of the π^±^–d system, the contribution is negligible^32,33^. To gauge the influence of the resonance decays on the π^±^–d correlations, the contributions of the Δ resonances extracted from the measured π^±^–p correlations are modified assuming that the nucleon emerging from the Δ decay coalesces with an additional nucleon to form a deuteron. The assumption is that the two nucleons have similar momenta (see Methods for details). Finally, the relative momentum k* between the pion from the Δ decay and the deuteron is evaluated. All charge states Δ^++,+,0,−^ are considered, assuming the Δ^+,−^ peak has the same shape as Δ^++,0^ from π^±^–p correlations. The experimental correlation functions are well described, confirming the scenario in which the deuteron is formed after the decay of the Δ by a fusion process as assumed in Fig. 1c. An excellent description (Fig. 2) of the measured correlation function is obtained by adopting the data-driven shape of the Δ derived from π^±^–p correlations^38^. The Δ shape in the π^±^–d correlation function exhibits a shift towards lower masses due to rescattering effects, consistent with the displacement observed in Fig. 2 relative to the nominal Δ position at k* ≈ 240 MeV *c*^−1^. The simulations obtained with the EPOS 3 event generator at present do not include the rescattering of the Δ decay products, resulting in no shift of the Δ peak in Fig. 1c.

The evidence of Δ decay in the π^±^–d correlation function is model-independent, as freeing the radius parameter does not affect the results, the Coulomb interaction is inherently model-independent and the residual background is accounted for in the systematic uncertainties of the fit.

Furthermore, the fraction of deuterons produced following a resonance decay is extracted. The contribution from Δ resonances is evaluated by integrating the peak in the π^±^–d correlation functions corresponding to these resonances, subtracting the number of π^±^–d pairs expected in the same k* region without a nucleon originating from the Δ resonance (Coulomb + background) and dividing the result by the total number of detected deuterons. The result is corrected for combinatorial effects, reconstruction efficiency and the non-measured π^0^ final state. With these corrections, the fraction of deuterons produced by a Δ resonance is calculated to be 60.6 ± 4.1% (Methods). To test the compatibility of this measurement with expectations from event generators, the EPOS 3 model is used. For this, the yield of baryonic resonances in EPOS 3 was adjusted to match the predictions of the CSM ThermalFIST for pp collisions at √*s* = 13 TeV. Furthermore, the change in the pion detector acceptance resulting from the shift in the experimentally observed Δ spectral shape is taken into account. In this simulation, the fraction of deuteron for which at least one of the nucleons stems from any Δ resonance and both the deuteron and the π are found within the acceptance is determined to be 53.8 ± 3.1% (more details in the Methods). The fact that these two fractions are in agreement within 1.32 standard deviations demonstrates that the survival probability of deuterons produced in pp collisions at the LHC is very high. This is the case because (anti)nuclei are produced after the resonance decays, in which spectral temperatures of about 20 MeV (see the Methods for details) have been evaluated, much lower than the average kinetic energy of hadrons (about 100 MeV) in pp collisions at the LHC.

Deuterons can form through fusion after any strong resonance decay, with Δ resonances accounting for 77.3 ± 1.2% of all cases. Moreover, considering that 15.5 ± 0.5% of Δ resonances are not properly reconstructed because of the detector acceptance effects, the experimental fraction of deuterons from Δ can be scaled up to a total fraction of deuterons originating from all resonances of 88.9 ± 6.3%. These results indicate not only that the presence of resonances can contribute to the (anti)deuteron production but also that it is the dominant process responsible for the creation of deuterons.

## Summary

In this work, π^±^–d correlation functions measured in pp collisions at √*s* = 13 TeV by the ALICE Collaboration at the LHC are used to study the (anti)deuteron production mechanism. It is demonstrated that (anti)deuteron formation by nucleonic fusion follows the strong decay of short-lived resonances. Model-independent evidence is provided by observing the residual correlation of pion–nucleon pairs stemming from the same Δ decay in the pion–deuteron correlation function. This effect can be explained only assuming that (anti)deuteron formation occurs after the Δ decay and the measured correlation is interpreted by a data-driven method based on the independent measurement of the Δ in the π^±^–p final state. The residual signal in the π^±^–d correlations can be used to evaluate the fraction of (anti)deuterons produced following Δ decays, which is found to be 60.6 ± 4.1%. Extending this reasoning to all strong resonances produced in pp collisions at √*s* = 13 TeV, it is found that 88.9 ± 6.3% of (anti)deuterons are formed through binding processes involving nucleons originating from strongly decaying resonances. This large fraction demonstrates that most of the (anti)nuclei are produced through secondary binding processes in pp collisions at the LHC and not by direct emission as other hadrons. A large survival probability is expected for (anti)deuterons as the low spectral temperature of Δ (about 20 MeV) reflects that the environment in which (anti)nuclei are created is characterized by a much lower kinetic energies than the hadronization phase (around 100 MeV) in pp collisions at the LHC. These findings solve a longstanding puzzle in nuclear physics, providing insight into the microscopic mechanism that leads to (anti)nuclei formation in pp collisions at the LHC. These insights can now be used for a more realistic microscopic modelling of (anti)nuclei production, for example, in reactions induced by cosmic ray.

## Methods

### Event selection

The results are based on the analysis of a dataset comprising inelastic pp collisions at √*s* = 13 TeV, recorded with the ALICE detector^45,46^ during the LHC run 2 (2015–2018). The events are selected using a high-multiplicity (HM) trigger, which captures the highest multiplicity events—specifically, the top 0.17% of all inelastic collisions that include at least one charged particle within the pseudorapidity interval |*η*| < 1 (denoted as 0.17% INEL > 0). This approach ensures a statistically rich sample, as a five-fold increase in the production of (anti)deuteron candidates has been observed in HM pp collisions compared with minimum bias pp collisions^47^. The sample of HM-triggered collisions considered for this analysis corresponds to 1 × 10^9^ events. On average, 31 charged tracks are found within |*η|* < 0.5 (ref. ^48^) for the HM-triggered collisions. Detailed descriptions of the event selection criteria, pileup rejection techniques, primary-vertex reconstruction methods and the HM trigger procedure are provided in ref. ^49^.

### Tracking and particle identification

Particle identification and momentum measurement of charged particles are performed using the inner tracking system (ITS)^50^, time projection chamber (TPC)^51^ and time-of-flight (TOF)^52^ detectors of ALICE covering the whole azimuthal angle and the pseudorapidity interval |*η*| < 0.9. These detectors are located within a uniform magnetic field of 0.5 T along the beam axis, generated by the ALICE solenoid magnet, which causes the trajectories of particles to bend. The curvature of the charged-particle tracks is used to measure the particle momenta. The transverse momentum for pion and deuteron candidates is determined with a resolution ranging from approximately 2% for tracks with *p*_T_ ≈ 10 GeV *c*^−1^ to below 1% for *p*_T_ < 1 GeV *c*^−1^. Particle identification is performed by measuring the energy loss per unit track length (d*E*/d*x*) in the TPC detector and the particle velocity (*β*) in the TOF detector. For tracks in the TPC detector, the signal is obtained from the *nσ*_TPC_ distribution, where *nσ*_TPC_ represents the deviation of the measured signal from the expected value for a given particle hypothesis, normalized by the detector resolution. Similarly, for the TOF detector, the resolution is defined by *nσ*_TOF_, which quantifies the difference between the measured and expected time of flight, also normalized by the resolution. Further experimental details are discussed in ref. ^46^. The selection criteria for pion and deuteron tracks used in this work are described in refs. ^30,41^.

Pions are identified by the measurement of the specific energy loss within |*nσ*_TPC_| < 3 in a transverse momentum range *p*_T_ ∈ [0.14, 4.0] GeV *c*^−1^. This information is combined with the TOF measurement by taking the geometric sum, $\sqrt{n{\sigma }_{{\rm{TPC}}}^{2}+n{\sigma }_{{\rm{TOF}}}^{2}} < 3$, for track momentum *p* > 0.5 GeV *c*^−1^. Similarly, the deuteron candidates are selected within a transverse momentum range *p*_T_ ∈ [0.8, 2.4] GeV *c*^−1^. They are identified by using |*nσ*_TPC_| < 3 for candidate tracks with momentum *p* < 1.4 GeV *c*^−1^, whereas both TPC and TOF information are required, $\sqrt{n{\sigma }_{{\rm{TPC}}}^{2}+n{\sigma }_{{\rm{TOF}}}^{2}} < 3$, for candidates with *p* > 1.4 GeV *c*^−1^. Moreover, for (anti)deuteron candidate selections, electrons are rejected by the condition *nσ*_TPC,e_ > 6 for *p* < 1.4 GeV *c*^−1^ and pions are rejected by the condition *nσ*_TPC,π_ > 3 for the tracks with momentum *p* > 1.4 GeV *c*^−1^. Overall, using these methods, a purity of 99% for π^±^ and 100% for (anti)deuterons is achieved.

The selection criteria of pions and deuterons constitute the primary source of systematic uncertainties associated with the measured correlation function. All particle selection criteria are varied from their default values. To account for the effect of possible correlations, the analysis of π^+^–d and π^−^–d pairs is repeated 44 times using random combinations of these selection criteria. The total systematic uncertainties are extracted by first randomly selecting a correlation function from the 44 systematic variations. For each sampled function, a bootstrap method is applied by randomly varying the *C*(k*) values in the individual k* bins according to their statistical uncertainties, assuming Gaussian errors. This results in a distribution of values for each k* bin, which is then fitted to determine the total uncertainty. As the statistical and systematic uncertainties are independent, the total uncertainty is obtained by adding them in quadrature. The systematic component is then determined by subtracting the known statistical uncertainty. The systematic uncertainties are largest at low k* ≈ 10 MeV *c*^−1^, reaching 1%. The same procedure is applied to extract the uncertainties of the fitted parameters and propagated to the final results on the fraction of deuterons stemming from resonance-assisted fusion processes.

### Characterization of the particle-emitting source

A standard approach to evaluate the source function, used by ALICE in pp collisions, is the resonance source model (RSM)^41,44^. In these publications, the ALICE Collaboration measured the source size for baryon–baryon, meson–baryon and meson–meson pairs, demonstrating a common emission source of all particles and resonances produced directly in the collision. These are described as primordial particles, whereas the short-lived resonances that decay into the pairs of interest on the timescale of fm *c*^−1^ will lead to an increase in the effective source size. If this increase in the source size is properly modelled by Monte Carlo simulations, the underlying primordial source has a Gaussian profile of width *r*_core_, and scales as a function of the pair transverse mass ${m}_{{\rm{T}}}=({k}_{{\rm{T}}}^{2}+{m}^{2}{)}^{1/2}$, where *m* is the average mass, the average of the masses of the two particles constituting the pair and *k*_T_ =  |**p**_T,1_ + **p**_T,2_|/2 is the average transverse momentum of the pair^41,44^. The scaling of the primordial source size follows a power law ${r}_{{\rm{core}}}=a{\langle {m}_{{\rm{T}}}\rangle }^{b}+c$, where the parameters for the high-multiplicity pp collisions at √*s* = 13 TeV used for the present π–d analysis are provided in ref. ^44^. The knowledge of both the pair average *m*_T_ and the cocktail of contributing resonances allows us to evaluate both the *r*_core_ and subsequently the total source distribution *S*(r*). The present analysis incorporates the resonances decaying into pions from the ThermalFIST model^34,37^, as already performed in the ALICE π–π and p–π analyses^38,41^. From the study of p–d and K^+^–d correlations in pp collisions at √*s* = 13 TeV (ref. ^30^), it has been shown that in pp collisions, the hadron–deuteron pairs follow the same transverse mass scaling as other hadron–hadron pairs, allowing to constrain the π–d emission source using the RSM. The deuterons are not produced directly by resonances. Nevertheless, the present work demonstrates that resonances decaying into nucleons are an important step in the production mechanism. This will lead to an effective delay in the deuteron production, an effect already described in a previous analysis of the K^+^–d analysis^53^. The present analysis adopts a conservative approach and integrates two extreme scenarios for the deuteron production as part of the systematic uncertainties, namely, assuming either that all deuterons are primordial or that the deuteron formation is delayed based on the amount of emission delay by which their constituent nucleons are affected^44^. This variation, which affects the effective source size, *r*_eff_ of up to 0.08 fm, is included in the systematic uncertainties on the modelling of the correlation functions. The final values for the *r*_eff_, after the inclusion of resonances, are summarized in the Extended Data Table 1, along with the total uncertainties.

### Corrections of the correlation function

The experimental correlation function, defined as $$C({k}^{* })=$$$${\mathcal{N}}\,[{N}_{{\rm{same}}}({k}^{* })/{N}_{{\rm{mixed}}}({k}^{* })]$$ is only corrected by a normalization constant $${\mathcal{N}}$$, by ensuring that the correlation becomes unity for k* ∈ (400, 600) MeV *c*^−1^. The remaining corrections are included in the fit function

1 $${C}_{{\rm{fit}}}({k}^{* })=\varepsilon ({k}^{* })\otimes B({k}^{* })[{\lambda }_{{\rm{gen}}}{C}_{{\rm{gen}}}({k}^{* })+(1-{\lambda }_{{\rm{gen}}})].$$

The parameter *ε*(k*) incorporates momentum resolution effects, which are included by obtaining a transformation matrix that can be used to apply resolution effects to the correlation functions. Details on the procedure are provided in the supplemental materials in ref. ^44^. The required experimental inputs are the matrix itself and the experimental mixed-event sample, both of which are provided in the HEPData entry related to this work. The baseline $B({k}^{* })=a+b{k}^{* 2}+c{k}^{* 3}$ accounts for any remaining long-range correlations^54^. These correlations do not contribute as an additive contamination to the correlations as misidentified particles do, but rather stem from the kinematics of the collision event. These long-range correlations are not correlated to the final-state interaction and can therefore be factorized and included as a multiplicative factor in the correlation. All the parameters of the baseline are left free in the fit procedure. The final correction to the correlation function is *λ*_gen_, which represents the amount of genuine π–d pairs. In the context of the source, a genuine particle is either a primordial or the decay product of a short-lived resonance of lifetime *cτ* < 5 fm *c*^−1^. Details on the extraction of these parameters for the pions and deuterons are provided in ref. ^41^ and ref. ^30^, respectively. Combining the information for the two species, the correction obtained for π^±^–d is summarized in Extended Data Table 1. The (1 − *λ*_gen_) factor in the definition of *C*_fit_(k*) reflects the remaining non-genuine correlations, which are assumed to produce a flat correlation signal. These non-genuine correlations stem from misidentified particles, as well as feed-down from long-lived resonances. Owing to the high purity in the present analysis, the non-genuine correlations are predominantly linked to the feed-down into pions from non-strong decays, such as decays of kaons^41^. There is no contribution to the non-genuine correlation from feed-down into deuterons, as these decay processes do not exist, except for the weak decay of the hypertriton $(\genfrac{}{}{0ex}{}{3}{\Lambda }{\rm{H}}\to {{\rm{\pi }}}^{-}+{\rm{p}}+{\rm{d}})$, which has a negligible effect.

### Spectral shape of Δ

In the measurement of the π^±^–p correlation functions^38^, a prominent peak around k* = 211 MeV *c*^−1^ can be seen, associated with the Δ resonances (Δ^++^ for π^+^–p and Δ^0^ for π^−^–p). In the decay of a Δ resonance into a pion–nucleon pair, the Δ is at rest in the centre-of-mass frame of the decay products. By applying energy and momentum conservation for this two-body decay, the invariant mass of the Δ is related to the relative momentum k* of the decay products by

2 $${m}_{\Delta }=\sqrt{{({k}^{* })}^{2}+{m}_{{\rm{\pi }}}^{2}}+\sqrt{{({k}^{* })}^{2}+{m}_{{\rm{N}}}^{2}}.$$

Inverting this expression yields the expected relative momentum k* associated with a given Δ mass

3 $${k}^{* }=\frac{\sqrt{{({m}_{\Delta }^{2}-{m}_{{\rm{\pi }}}^{2}-{m}_{{\rm{N}}}^{2})}^{2}-4{m}_{{\rm{\pi }}}^{2}{m}_{{\rm{N}}}^{2}}}{2{m}_{\Delta }}.$$

For the nominal Δ mass of *M*_Δ_ = 1.215 GeV *c*^−^^2^, this corresponds to a relative momentum of k* = 211 MeV *c*^−1^ for the pion–nucleon pair. The peak position observed in the π^±^–p correlations is shifted to lower values than the nominal Δ mass because of the rescattering of the decay products and regeneration of the resonances^55–57^.

Following ref. ^38^, the Δ spectral shape is modelled as ${C}_{\Delta }({k}^{\ast })={{\mathcal{N}}}_{\Delta }\times PS({p}_{{\rm{T}},\Delta },T)\times Sill({M}_{\Delta },{{\Gamma }}_{\Delta })$. The first term ${{\mathcal{N}}}_{\Delta }$ is a normalization constant, whereas the undisturbed spectral shape of the resonances is described usingthe Sill distribution^58^, which depends on the resonance mass *M*_Δ_ and width *Γ*_Δ_. As the *Sill* is expressed as a function of k*, it is essential to account for the Jacobian factor $| {\rm{d}}{m}_{\Delta }/{\rm{d}}{k}^{* }|$ in the change of variables, where *m*_Δ_ is given by equation (2). Modifications of the spectral shape due to rescattering and resonance regeneration effects are incorporated by a multiplicative *PS*(*p*_T,Δ_, *T*) term^56,57^, a Boltzmann-like phase space factor,

4 $$PS({p}_{{\rm{T}},\Delta },T)\propto \frac{M}{\sqrt{{M}^{2}+{p}_{{\rm{T}},\Delta }^{2}}}\exp \left[-\frac{\sqrt{{M}^{2}+{p}_{{\rm{T}},\Delta }^{2}}}{T}\right],$$

acting as a weight for the emission of the resonance with certain transverse momentum *p*_T,Δ_ at a temperature *T*. The latter is referred to as the ‘Δ spectral temperature’ in ref. ^38^.

To obtain the corresponding spectral shape in the π–d correlation, a simple approach is adopted by assuming that each measured deuteron consists of two nucleons of equal momenta. In this way, the Δ spectral shape in the π–N system (equation (3)) can be transformed into the π–d PRF. For a nominal Δ mass of *M*_Δ_ = 1.215 GeV *c*^−^^2^ leads to k* = 237 MeV *c*^−1^ in the pion–deuteron system.

A final systematic check was performed by allowing a non-zero relative momentum between the two nucleons forming a deuteron. For this, a relative momentum sampled from a distribution, which was obtained from a coalescence model^59^, was used. The relative momentum is, on average, about 100 MeV *c*^−1^ (ref. ^59^). The final shape of the Δ peak in the π–d correlation remains identical regardless of the assumption of the relative momenta between the nucleons. Thus, the simpler approach of identical nucleon momenta was used in the analysis.

### Fitting the π–d correlation

The fit function is defined by equation (1). The genuine correlation *C*_gen_(k*) encapsulates Coulomb and strong interactions alongside contributions from the Δ resonance. The interaction components were modelled using the CATS framework^42^, which uses the Schrödinger equation and requires as input the source function and the strong interaction potential. The contribution of the strong interaction is minimal because of the small scattering parameters of the π–d system, as the scattering length is cancelled for π–p and π–n pairs^32,33^. The real part of the π^−^–d potential was included in the fit^32,33^. To account for the Δ resonance, a phenomenological approach was adopted, expressing the genuine correlation as

5 $${C}_{{\rm{gen}}}({k}^{* })={C}_{{\rm{interaction}}}({k}^{* })[{F}_{\Delta }{{\mathcal{A}}}_{\Delta }{C}_{\Delta }({k}^{* })+(1-{F}_{\Delta }{{\mathcal{A}}}_{\Delta })],$$

where *F*_Δ_ is a free parameter representing the number of Δ resonances contributing to deuteron production divided by the number of all measured deuterons. The parameter ${{\mathcal{A}}}_{\Delta }$ is an arbitrary normalization constant, introduced to keep the physically motivated definition of *F*_Δ_ intact. The term *C*_Δ_(k*) reflects the spectral shape of the Δ resonance measured and fitted in the π–p analysis by ALICE (see previous section)^38^, transformed to the π–d system. The mass (*M*_Δ_ = 1,215 MeV *c*^−^^2^) and width (*Γ*_Δ_) of the Δ resonance in the present analysis are fixed to the values extracted from the measured π–p correlations, whereas the Δ spectral temperature *T* is fitted. The width *Γ*_Δ_ is dependent on *m*_T_, for the *m*_T_-integrated data shown in Fig. 2, the value is 95 MeV *c*^−^^2^.

The fit to the data is performed in the range k* ∈ (0, 500) MeV *c*^−1^, with a systematic variation of k* ∈ (0, 600) MeV *c*^−1^. As a systematic check, a 5% variation in *λ*_gen_ is considered, accounting for the uncertainties arising in the determination of secondary contributions and purities due to systematic variations in the particle candidate selection criteria. As the parameters *F*_Δ_ and ${{\mathcal{A}}}_{\Delta }$ are maximally correlated, the fit is performed using the effective parameter ${F}_{\Delta }^{{\prime} }={F}_{\Delta }{{\mathcal{A}}}_{\Delta }$. The parameter ${F}_{\Delta }^{{\prime} }$ represents the fraction of π–d pairs in which the pion and at least one of the nucleons within the deuteron originate from a Δ. This can be expressed as

6 $${F}_{\Delta }^{{\prime} }=\int {C}_{\Delta }({k}^{* }){N}_{{\rm{mixed}}}({k}^{* }){\rm{d}}{k}^{* }\,/\,\int {N}_{{\rm{mixed}}}({k}^{* }){\rm{d}}{k}^{* }.$$

As the key parameter in this study is *F*_Δ_, establishing a relationship with ${F}_{\Delta }^{{\prime} }$ is necessary. A straightforward analytical transformation can be derived under the assumption that most recorded collisions containing a reconstructed deuteron include only one. This implies that no additional Δ signal is introduced in the peak region because of combinatorial effects, and the number of Δ resonances associated with deuteron production becomes equal to the number of pairs (peak amplitude) linked to a Δ. This results in

7 $${F}_{\Delta }\approx \int {C}_{\Delta }({k}^{* }){N}_{{\rm{mixed}}}({k}^{* }){\rm{d}}{k}^{* }/{N}_{{\rm{d}}},$$

where *N*_d_ is the total number of reconstructed deuterons used in the analysis. Given the fraction of events containing more than one deuteron, the uncertainty associated to equation (7) is estimated to be negligible (≲0.03%). Using equations (6) and (7)

8 $${F}_{\Delta }=\frac{\int {N}_{{\rm{mixed}}}({k}^{* })}{{N}_{{\rm{d}}}}{F}_{\Delta }^{{\prime} }=0.533\pm 0.035,$$

where both *N*_mixed_(k*) and *N*_d_ are measured, whereas ${F}_{\Delta }^{{\prime} }$ is extracted from the fit. The quoted uncertainty combines the statistical and systematic errors of the data and the fit. The fit results for the phase space parameters (equation (4)) are *p*_T,Δ_ = 985 ± 171  MeV *c*^−1^ and *T* = 20 ± 2 MeV.

### Deuteron and proton fraction from resonances

Relating *F*_Δ_ to the probability *P*_Δ_ of producing a single nucleon from a Δ resonance requires accounting for the reconstruction efficiency. Although the efficiency of deuterons cancels out because of the definition of *F*_Δ_, the pion reconstruction efficiency, *ε*_π_, must be included. The pion efficiencies are obtained using Monte Carlo simulations produced with PYTHIA 8.2 (ref. ^60^), tuned to reproduce pp collisions at 13 TeV, and filtered through the ALICE detector and reconstruction algorithm^45^.

The following calculations are based purely on combinatorial considerations, without explicitly accounting for the microscopic or kinematical properties of the resonances. The probability of producing exactly one of the two nucleons within the deuteron from a Δ resonance and detecting the decay pion is 2*ε*_π_*P*_Δ_(1 − *P*_Δ_). The probability of having both nucleons within the deuteron originating from a Δ resonance and detecting both decay pions is ${\varepsilon }_{{\rm{\pi }}}^{2}{P}_{\Delta }^{2}$, whereas the probability for the same production scenario when failing to detect one of the pions is $2{\varepsilon }_{{\rm{\pi }}}(1-{\varepsilon }_{{\rm{\pi }}}){P}_{\Delta }^{2}$. Note that in the case in which both nucleons in the deuteron stem from a Δ, the final state contains a single deuteron and two pions, resulting in two entries in the peak region of the correlation function. As *F*_Δ_ is defined as the ratio of the number of π–d pairs to single deuterons, the corresponding term, ${\varepsilon }_{{\rm{\pi }}}^{2}{P}_{\Delta }^{2}$, contributes with twice the number of pairs. Taking all these considerations into account and adding all of the terms together leads to

9 $${F}_{\Delta }=2{\varepsilon }_{{\rm{\pi }}}{P}_{\Delta }.$$

Owing to the effect of double counting some of the pairs, the result must be transformed into the fraction of (single) deuterons, *f*_Δ_, produced by a Δ resonance. The definition of *f*_Δ_ is similar to *F*_Δ_, but it removes the double-counting effect by taking the pure term ${\varepsilon }_{{\rm{\pi }}}^{2}{P}_{\Delta }^{2}$ without additional multiplication by 2. This leads to the expression

10 $${f}_{\Delta }=2{\varepsilon }_{{\rm{\pi }}}{P}_{\Delta }(1-\frac{{\varepsilon }_{{\rm{\pi }}}{P}_{\Delta }}{2}).$$

Equations (9) and (10) account for the pion reconstruction efficiency, *ε*_π_, in correcting the single-particle purities. Consequently, *f*_Δ_ is evaluated after applying this efficiency correction. The efficiency-independent result, ${f}_{\Delta }^{{\rm{true}}}$, is obtained by setting *ε*_π_ = 1 and expressing it in terms of the measured *F*_Δ_

11 $${f}_{\Delta }^{{\rm{true}}}=2{P}_{\Delta }\left(1-\frac{{P}_{\Delta }}{2}\right)=\frac{{F}_{\Delta }}{{\varepsilon }_{{\rm{\pi }}}}\left(1-\frac{{F}_{\Delta }}{4{\varepsilon }_{{\rm{\pi }}}}\right).$$

Considering the experimental result *F*_Δ_ = 0.533 ± 0.035 and a pion reconstruction efficiency of *ε*_π_ = 71.53 ± 0.65%, evaluated using Monte Carlo simulation and averaged over the transverse momentum range for the pion candidates considered in the analysis, the true fraction is calculated as ${f}_{\Delta }^{{\rm{true}}}=60.6\pm 4.1 \%$. The uncertainty is propagated by treating the errors of *F*_Δ_ and *ε*_π_ as independent.

It should be noted that this value must be considered a lower limit, as it is possible for the pion from the Δ decay to escape the detector acceptance, while the associated nucleus is still reconstructed. This loss of Δ resonances can be estimated only in a model-dependent manner using Monte Carlo event generators. Using EPOS 3 and PYTHIA 8.3, the loss of Δ particles due to *η* acceptance effects of the pion is estimated to be 15.5 ± 0.5%, implying that the value of *F*_Δ_ is underestimated by a similar amount. This number is obtained by calculating the acceptance as a function of k* folded with the measured k* distribution of the delta resonance. Re-evaluating ${f}_{\Delta }^{{\rm{true}}}$ using equation (11) by including in addition the acceptance effect, the result becomes ${f}_{\Delta }^{{\rm{true}}}=68.7\pm 4.6 \%$.

Similar relations apply to deuterons produced from any resonance. However, the corresponding value is experimentally inaccessible because of the large spectral widths and small individual contributions of the other resonances. By defining the total fraction as *f*_R_ and assuming that the ratio *f*_Δ_/*f*_R_ = 0.773 ± 0.012, as predicted by the CSM models, holds for the experimental data, it is possible to extrapolate the acceptance corrected ${f}_{\Delta }^{{\rm{true}}}$ to ${f}_{{\rm{R}}}^{{\rm{true}}}=88.9\pm 6.3 \%$.

The fraction of deuterons from Δ resonances was also obtained using the EPOS event generator. For this, all resonances included in EPOS were reweighted using the CSM ThermalFIST with the settings shown in the Extended Data Table 2. The deuteron formation is simulated using a coalescence afterburner^25^ in which the information about the mother particles of the nucleons is conserved. For each nucleon in the deuteron, the potential resonance mother is identified and it is checked, whether the nucleon and the corresponding π fall within the *p*_T_ and *η* acceptance. If at least one nucleon in the deuteron fulfils this criterion, the deuteron is counted as stemming from a resonance. Finally, the *η*-acceptance of π is expected to be different in EPOS compared with the measurement, as the spectral shape of the Δ in the experiment is shifted towards lower k* values. Losses due to this *η*-acceptance increase for increasing k*, and thus are lower in reality than in the simulation. For this, the acceptance as a function of k* is averaged using the experimental Δ spectral shape. The resulting acceptance is 83.6%, whereas a similar study with PYTHIA 8.3 gives 85.4%. Averaging these values and taking the variance as an uncertainty, the Monte Carlo yields ${f}_{\Delta }^{{\rm{true,MC}}}=53.8\pm 3.1 \%$, a result compatible with the experimental value of 60.6 ± 4.1%.

Although removing the model dependence of this estimation is not feasible, a validation can be performed using π–p correlation measurements^38^. For this purpose, we define the experimental fraction

12 $${F}_{\Delta \to {\rm{p}},\exp .}={Y}_{\exp }(\Delta )/[{Y}_{\exp }({\rm{p}}){\varepsilon }_{{\rm{\pi }}}]$$

for the π–p correlation function, where *Y*_exp_ denotes the experimentally measured yields of Δ and proton candidates. *Y*_exp_(Δ) is derived from the spectral shape *R*_Δ_(k*) published in ref. ^38^, and the proton–pion mixed-event distribution ${N}_{{\rm{mixed}},{\rm{p}}-{{\rm{\pi }}}^{\pm }}$ using the relation

13 $${Y}_{\exp }(\Delta )=\int {R}_{\Delta }({k}^{* }){N}_{{\rm{mixed}},{\rm{p}}-{{\rm{\pi }}}^{\pm }}{\rm{d}}{k}^{* }.$$

This yields proton fractions of (14.2 ± 0.4)% from Δ^++^ decays and (5.8 ± 0.3)% from Δ^0^ decays. The performance of the Monte Carlo event generators has been validated by calculating the corresponding model-based fraction *F*_Δ→p,MC_, using ThermalFIST to fix the initial yields of resonances and baryons, followed by PYTHIA or EPOS simulations within the ALICE acceptance. The change in the *η*-acceptance of the pion due to the shift of the experimentally observed Δ spectral shape is accounted for as described above. With this, ThermalFIST + EPOS predicts that 13.4% of the protons stem from Δ^++^ within the experimental acceptance and 4.6% from Δ^0^. The corresponding numbers in ThermalFIST + PYTHIA are 14.6% for Δ^++^ and 5.0% for Δ^0^.

### Simulations

The simulation of the π^+^–d correlation function was performed for three different hypotheses. The first simulation (Fig. 1c) is done using the EPOS 3 event generator combined with a coalescence afterburner developed in ref. ^25^, and it is able to reproduce the total number of deuterons in the analysed dataset without any free parameters. The deuterons obtained from this coalescence afterburner are combined with all pions of the desired charge in the same event to create the same-event distribution and with a buffer of up to 50 pions from previous events to build the mixed-event distribution. The predictions using ThermalFIST use the ThermalFIST sampler^34,61^, which uses a Cooper-Frye particlization sampling procedure^61^ and a blast-wave parameterization^62^ tuned to pp collisions at √*s* = 13 TeV (ref. ^2^) to obtain positions and momenta of the particles. In the blast-wave model, a thermalized medium expands radially with a subsequent instantaneous freeze-out. Its main parameters are the average expansion velocity ⟨*β*⟩, its kinetic freeze-out temperature *T*_kin_ and the velocity profile exponent *n*. ThermalFIST can directly produce deuterons without the need for an afterburner, and the same mixed events can be directly constructed in a similar manner as before. The last prediction obtained with ThermalFIST and SMASH uses the particles output by the ThermalFIST sampler in the kinematic region |*η*| < 1, including deuterons, and feeds it into the hadronic afterburner SMASH^35^. Inside SMASH, particles rescatter for up to 15 fm *c*^−1^ with a fixed timestep of Δ*t* = 0.001 fm *c*^−1^. The stochastic collision criterion is chosen to enable deuteron production and destruction using the 3 ↔ 2 scattering processes such as p + n + π ↔ d + π. Furthermore, all 2 ↔ 2 processes included in SMASH are enabled. The parameters used in ThermalFIST and the blast-wave model are shown in Extended Data Table 2.

### Resonance spectral temperature

**Extended Data Fig. 1:**
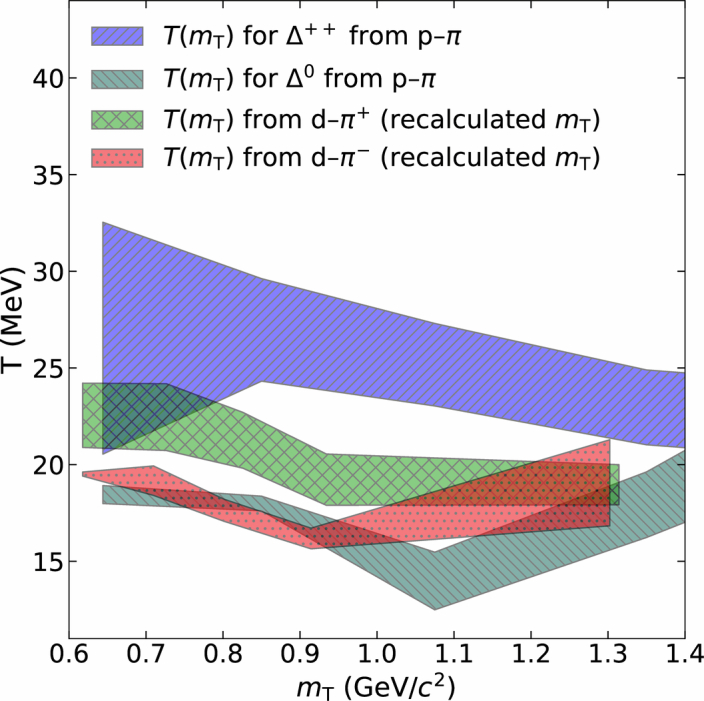
Extracted Δ spectral temperature. The Δ spectral temperature is derived from π^±^–p and π^±^−d correlation functions measured in high-multiplicity pp collisions at $\sqrt{{s}}$ = 13 TeV. The bands correspond to the uncertainties obtained by fits to the correlation functions, incorporating systematic uncertainties on the measured data, as well as those arising from variations in the source size and the *λ* parameter for the genuine interaction.

The modifications of the Δ spectral shape in the fitting procedure are modelled with the *PS* term presented in equation (4). As discussed above, this function is effectively controlled by the Δ spectral temperature *T*. The Δ spectral temperatures for the π^±^–d system are shown in the Extended Data Fig. 1 as a function of the transverse mass, *m*_T_, of one nucleon in the deuteron and the pion. They are comparable for π^+^–d and π^−^–d but differ from the π^±^–p systems discussed in ref. ^38^, being lower than the spectral temperature found for Δ^++^ and higher than that of Δ^0^. This aligns qualitatively with the resonance regeneration, Δ ↔ Nπ, and rescattering picture^56,57^. For π^+^–p, repulsive strong and Coulomb interactions stop Δ^++^ regeneration and rescattering earlier, whereas the attractive π^−^–p interaction allows extended Δ^0^ regeneration and rescattering, leading to a lower Δ spectral temperature. In π^+^–d, the signal arises from Δ^++^ → π^+^p (with subsequent fusion with a neutron) or Δ^+^ → π^+^n (with later fusion with a proton). As π^+^–p interactions are repulsive and π^+^–n interactions attractive^63^, Δ^+^ undergoes longer regeneration cycles than Δ^++^. This results in a lower spectral temperature than a pure Δ^+^^+^ as reflected in the data. Similarly, the π^−^–d system includes contributions from Δ^0^ → π^−^p (attractive) and Δ^−^ → π^−^n (repulsive). The shorter regeneration phase of Δ^−^ compared with Δ^0^ yields a higher temperature for π^−^–d than a pure π^−^–p system, again seen in the measurements.

### Dibaryon hypothesis and π–d correlations

Although no dibaryon states have been confirmed unambiguously (such as a bound NΔ), candidates have been proposed to explain anomalies in data reported by WASA-at-COSY^64^, ELPH^65^ and BGOOD^66^. In particular, the NΔ(2114) candidate was first observed by ELPH with a reported mass of *m*_2B_ = 2,140 MeV *c*^−^^2^, and later by BGOOD at *m*_2B_ = 2,114 MeV *c*^−^^2^. The expected imprint of such a state on the π^±^–d correlation can be evaluated using ThermalFIST. Assuming an extreme upper limit of 100% for the unknown π–d branching ratio, as much as 28.5 ± 1.5% of deuterons could originate from this decay. Adopting a more realistic branching ratio, estimated from the ratio of elastic to inelastic π–d cross–sections^67^, reduces this fraction to only 9.5 ± 0.5%, far below the 60.6 ± 4.1% estimated in the present study. Furthermore, if this small contribution is taken as a template, the fit to the data is incompatible with the observed correlation. When the amplitude is left unconstrained, the measured signal remains consistent with at most 1% of deuterons being produced through these decays. Hence, the experimental data strongly disfavour dibaryon decays as a notable source of the observed signal in the measured π^±^–d correlation.

## Online content

Any methods, additional references, Nature Portfolio reporting summaries, source data, extended data, supplementary information, acknowledgements, peer review information; details of author contributions and competing interests; and statements of data and code availability are available at 10.1038/s41586-025-09775-5.

## Figures and Tables

**Extended Data Table 1:**
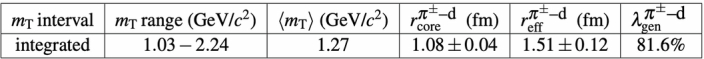
Parameters used in the measured correlation function

The values of the measured average transverse mass ⟨*m*_T_⟩, extracted source sizes ${r}_{{\rm{core}}}$, *r*_eff_, and *λ* parameter which serves as the weights for the contribution of the genuine π^+^ − d and π^−^ − d pairs in the measured correlation function.

**Extended Data Table 2:**
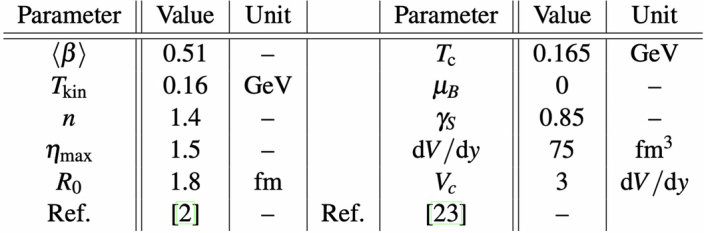
Parameters used in the π^+^–d correlation function predictions for thermal production

Parameters on the left side are used for the Blast-Wave parameterization, and parameters on the right are used for the ThermalFIST yields.

## Data Availability

This study has associated data in a HEPData repository at https://www.hepdata.net/record/ins2907586.

## Code availability

This study has associated code/software in a data repository. The code/software used for the analysis is publicly available at GitHub (https://github.com/alisw/AliRoot, https://github.com/alisw/AliPhysics and https://github.com/dimihayl/DLM/tree/master/CATS).
